# Developing SMS Content to Promote Papanicolaou Triage Among Women Who Performed HPV Self-collection Test: Qualitative Study

**DOI:** 10.2196/14652

**Published:** 2020-03-06

**Authors:** Victoria Sanchez Antelo, Racquel E Kohler, Mariana Curotto, Kasisomayajula "Vish" Viswanath, Melisa Paolino, Silvina Arrossi

**Affiliations:** 1 Centro de Estudios de Estado y Sociedad Buenos Aires Argentina; 2 Cancer Health Equity Cancer Institute of New Jersey Rutgers, The State University of New Jersey New Brunswick, NJ United States; 3 Programa Nacional de Prevención de Cáncer Cervicouterino Instituto Nacional del Cáncer Buenos Aires Argentina; 4 Department of Social and Behavioral Sciences Harvard T H Chan School of Public Health Harvard University Boston, MA United States; 5 McGraw-Patterson Center for Population Sciences Dana-Farber Cancer Institute Boston, MA United States; 6 Consejo Nacional de Investigaciones Científicas y Técnicas Buenos Aires Argentina

**Keywords:** text messaging, cell phone use, telemedicine, human papillomavirus DNA tests, triage, health behavior, Argentina

## Abstract

**Background:**

SMS interventions are effective in promoting a variety of health behaviors; however, there is limited information regarding the use of SMS for cervical cancer screening and follow-up care. The Application of Communication and Information Technologies to Self-Collection study aims to evaluate a multicomponent mobile health intervention to increase triage adherence among women with human papillomavirus (HPV)–positive self-collected tests in Jujuy, Argentina. Here, we describe the formative results used to design the content of the SMS to be tested in the trial.

**Objective:**

This study aimed to understand the cultural and contextual elements, women’s beliefs, and perceptions regarding the use of SMS by the health care system and women’s preferences about the message content.

**Methods:**

We conducted five focus groups (FGs), stratified by rural or urban residence and age. All participants were aged 30 years or older and had performed HPV self-collection. Participatory techniques, including brainstorming, card-based classification, and discussions were used to debate the advantages and disadvantages of messages. We openly coded the discussions for agreements and preferences regarding the SMS content. Messages for both HPV-negative and HPV-positive women were validated through interviews with health authorities and 14 HPV-tested women. The final versions of the messages were pilot-tested.

**Results:**

A total of 48 women participated in the FGs. Participants rejected receiving both negative and positive HPV results by SMS because, for them, the delivery of results should be done in a face-to-face interaction with health professionals. They stressed the importance of the SMS content informing them that results were available for pick up and reflecting the kind of relationship that they have with the community health workers and the nearest health center. Women considered that a personalized SMS was important, as was the use of a formal yet warm tone. Owing to confidentiality issues, not using the word “HPV” was also a key component of the desired SMS content; therefore, the final message included the term “self-collection” without the mention of HPV infection. Results from the validation stage and pilot test showed high acceptability of the final version of the message.

**Conclusions:**

The results suggest that SMS is accepted when notifying women about the availability of the HPV test result, but it should not replace the delivery of results in face-to-face, doctor-patient encounters. In addition, messages must be tailored and must have a persuasive tone to motivate women to adhere to the triage.

## Introduction

### Background

In Latin America, the high mortality rate of cervical cancer (CC) is related to problems with continuity in the screening process, including low participation in screening and abandonment of follow-up care procedures [1,2]. In recent decades, the development of the human papillomavirus (HPV) test has changed the screening paradigm: the HPV test has high sensitivity and negative predictive value [3,4] and has been demonstrated to reduce the incidence of CC and mortality [3]. Importantly, HPV testing allows for self-collection (SC), a method that is effective in detecting precancerous lesions [5] and has the potential to reduce barriers to screening, especially among underserved women [1,4,6,7].

SC is highly accepted by women in several countries, and studies have demonstrated that SC increases screening coverage [6,8-10], especially when the test is offered door-to-door by community health workers (CHWs) [6,11]. However, SC introduces an obstacle: women who test positive for HPV must undergo triage tests to identify those who must be referred for diagnosis and treatment. Although several triage methods are available for detecting precancerous lesions, cytology has been validated in several randomized trials [12] and is part of the screening policy recommended by the World Health Organization [13].

Using cytology as triage implies an additional appointment at the health center, which increases the risk of abandonment of follow-up care. A high triage adherence can be difficult to achieve in real-life programmatic contexts [14,15], especially among underscreened women [5,7]. In Argentina, 34% of HPV-positive women who performed SC at home during a CHW’s visit completed follow-up within 120 days after screening [14,15]. Studies that have analyzed adherence to different follow-up steps after abnormal cytology [15-18] showed that the delivery of test results presented an obstacle: women either did not receive or did not pick up the test results. In Argentina, not receiving SC results was one of the most reported barriers to follow-up care by HPV-positive women who were not adherent to the triage [15]. A separate study conducted in the context of cytology-based screening in Argentina also found that not receiving results was one of the most reported barriers to follow-up care by women who were not adherent to diagnosis and treatment [17].

In Jujuy, Argentina, where door-to-door HPV SC has been implemented since 2014, triage adherence increased to 77% in 12 months after a significant effort by CHWs to contact HPV-positive women in their homes [14]. However, home visits to all HPV-positive women as a public health strategy is difficult to sustain because of the high proportion of screened women who would need to be contacted (approximately 13%) [7]. In addition, CHWs are a scarce resource, and CC prevention is one of the many health services they provide to the population. In this sense, it is crucial to develop innovative strategies to improve the delivery of results and increase the adherence of HPV-positive women to the triage.

Various studies have shown that mobile health (mHealth) interventions are effective in changing health behaviors, such as following doctors’ recommendations, and in strengthening communication between users and health care professionals [19-23]. The use of text or SMS is the most frequently used mHealth strategy. Owing to its low cost, accessibility, and simple technology, SMS is appropriate for low- to middle-income settings [24-26]. In addition, specifically with respect to CC prevention, evidence has suggested that SMS-based interventions might increase screening uptake [24,27,28]. SMS interventions are accepted by women, and they have been shown to increase screening uptake among women who face cultural obstacles [29]. Hence, SMS could be used as a strategy to increase adherence to Papanicolaou (Pap) triage, without increasing the workload of CHWs (eg, door-to-door notification of availability of HPV SC results).

Despite its advantages, sending an SMS about a sexually transmitted infection (STI) such as HPV is complex. Patients’ privacy and confidentiality need to be protected and at the same time, the message must be short, easy to understand, and also culturally appropriate. In addition, it is important to use keywords to make the content clear without upsetting the recipient [30-32]. A study conducted in Chile about the SMS preferences of underscreened women showed that clarity and simplicity of the received message were very important for them [33]. The legitimacy of the sender and privacy issues, such as disclosing a result to a third person in shared cell phones, have also been shown to be relevant topics on SMS content [34,35]. Not taking into account the patients’ opinions about the SMS content, its design, and validation before the implementation of mHealth strategies have been pointed out as obstacles to the acceptability, effectiveness, and scalability of SMS-based health interventions [36-38].

### Objectives

Therefore, the objective of this study was to understand women’s beliefs and perceptions regarding the use of SMS in health care and their preferences regarding the message content, in addition to collecting data on cultural and contextual aspects. This formative research is part of a larger trial—the ATICA study (Application of Communication and Information Technologies to Self-Collection, for its initials in Spanish), a hybrid type 1 cluster randomized trial conducted in Jujuy, Argentina. The trial will evaluate whether SMS sent to HPV-positive women increases Pap triage among HPV-positive women with self-collected tests [39]. Results from the ATICA trial on the effectiveness of the use of SMS will be published in forthcoming papers. In this paper, we present the results from the formative research conducted to design the content of the SMS messages sent to women.

## Methods

### Theoretical Foundation

The methodological design of the study has been described elsewhere [39]; in brief, it involves sending SMS messages to HPV-positive women who have performed SC offered by CHWs during home visits. When women do not adhere to Pap triage within 60 days of a positive HPV test result, an email and SMS will be sent to the CHWs to alert them to visit the HPV-positive women who have not responded to reminders and encourage follow-up. The ATICA study is conceptually guided by the health belief model (HBM) [40,41], a framework that has been extensively used to explain cervical screening–related behaviors [42-45]. The HBM has 6 constructs: (1) *perceived susceptibility* of getting a disease or condition, (2) feelings about the seriousness of contracting it (*perceived severity*), (3) *perceived barriers* to address a recommended behavior to prevent or treat the disease, (4) the individuals’ evaluation about the *benefits-costs* of doing it, (5) the confidence in one’s ability to attend a health issue (*perceived self-efficacy*), and finally, (6) the *cues to action*, that is, external factors that potentiate the readiness to follow a new behavior [40]. Following the HBM, sending an SMS would work as a cue to promote actions and therefore prompt HPV-positive women to undergo Pap triage. In this sense, the SMS must address beliefs, values, and shared perceptions to encourage the prevention behavior [31,46,47].

We created a semistructured moderator guide for focus groups (FGs), including sections on (1) cell phone and SMS use, (2) dimensions of the HBM regarding SC and Pap triage, (3) perceptions and opinions about women’s relationship with the health system, (4) barriers for Pap triage, and (5) SMS content.

We used an array of participatory methods to create the SMS content [48,49]. The steps proposed by Abroms et al [37] were taken into account to create a health communication strategy oriented toward prompting behavior change via SMS. Following other proposals, we used a card sorting technique to debate predesigned content with the participants [32,49].

Following Muench and Baumel [32], SMS was divided into the following five structural elements:

- Greetings: terms of address that abide by the rules of etiquette in each cultural context and suitability for SMS communication in Spanish [50,51]
- Sender: source of the message and the authority that legitimizes the content [30]
- Message topic: preferences on how to refer to HPV SC
- Recipient: advantages and disadvantages of including the recipient’s name
- Closing and cue to action: purpose of the message of either notifying the results (HPV-negative) or availability of the SC results (HPV-positive)

**Figure 1:**
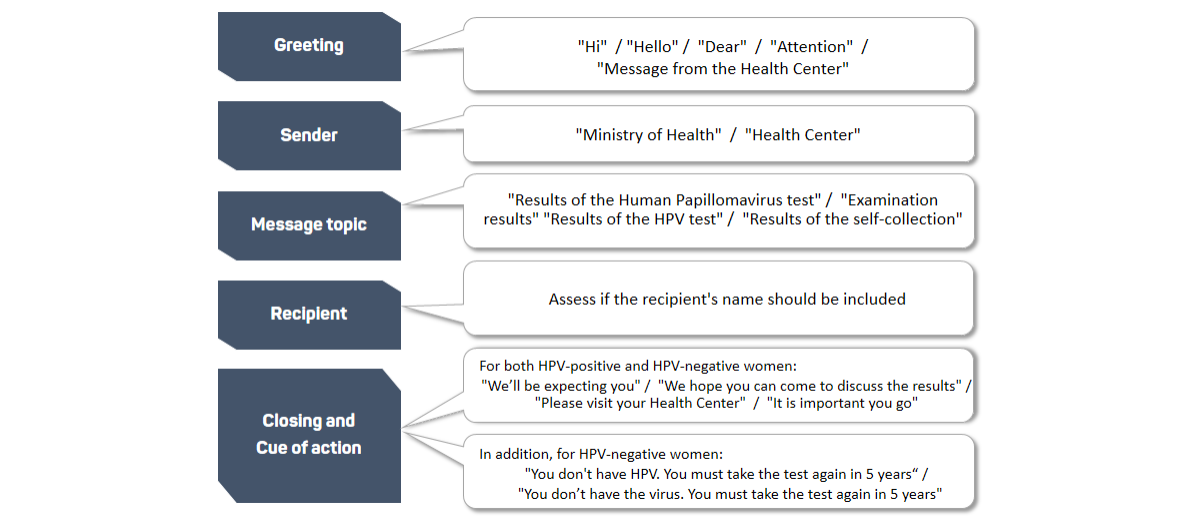
Different versions of the structural elements of the SMS used in focus groups. HPV: human papillomavirus.

Figure 1 shows different options for the five structural elements that were discussed by women.

Thereafter, different versions of each structural element were combined, which resulted in a total of 37 predesigned SMS messages (16 for HPV-negative results and 21 for HPV-positive results). Owing to the SMS restrictions, if the message combination was more than 140 characters, it was excluded from the research. Figure 1 shows different versions of the structural elements of the SMS messages used during FGs.

Finally, during FGs, the SMS content was determined through 3 activities: (1) brainstorming, which allowed spontaneous suggestions by participants, (2) pile sort method and ranking, in which participants sorted predesigned SMS (HPV-negative and HPV-positive) depending on whether they liked the content or not and then selected two cards from each pile (two most liked and two least liked), and (3) group debate, wherein each participant shared her motives for choosing each card.

**Figure 2:**
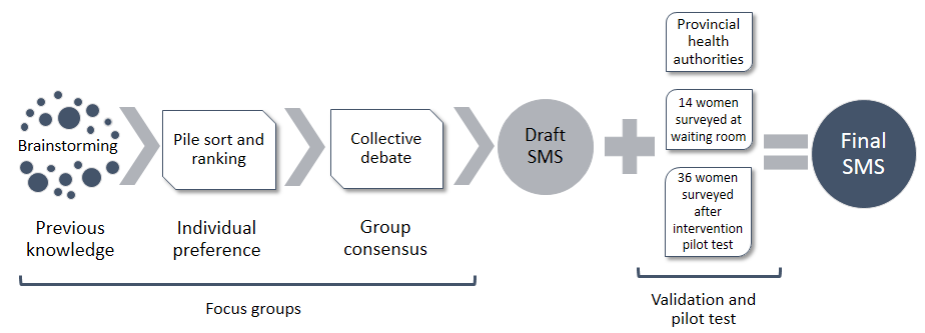
Stages of the SMS content design.

On the basis of the results from the FGs, 2 messages were designed and subjected to a double process of validation: (1) with the provincial health authorities (Primary Health Care Department and Program on Cervical Cancer Prevention) and (2) with the recipients, by means of a pilot survey to 14 women with characteristics similar to those of the FG participants. The survey included an assessment of the message comprehension, content appropriateness (words and tone), and perception of efficiency in increasing Pap triage (see Figure 2).

### Setting

According to the latest available data (2010), 673,307 people lived in Jujuy (343,387/673,307, 51.00% of them are women; 587,530/673,307, 13.00% of the people lived in rural areas). Furthermore, 3.00% (20,199/673,307) of the Jujuy population aged 10 years or older was illiterate, of which 68.60% (13,857/20,199) were women. Overall, 45.20% (304,335/673,307) of the total population of the province had public health insurance. In 2018, 30% of the urban population of the capital city (San Salvador-Palpalá) lived below the poverty line [52].

HPV testing has been the primary screening method for the prevention of CC since 2012 [7], targeting women aged 30 years and older who attend the public health system. HPV samples are collected by clinical staff at health centers. The screening protocol in use in Jujuy has been described elsewhere [7], but succinctly, HPV-positive women are triaged with cytology and those with the finding of atypical squamous cells of undetermined significance or worse, are further referred to colposcopy and biopsy if needed. Women with histologically confirmed cervical intraepithelial neoplasia grade 2 or worse are referred for treatment. HPV-negative women are recommended rescreening in 5 years. When HPV testing is done at health centers, HPV testing and cytology triage are conducted simultaneously, but cytology is read only if the HPV test is positive. Since 2014, HPV SC offered by CHWs during home visits was introduced as a programmatic strategy to increase screening coverage. In total, around 700 CHWs visit approximately 110,000 households twice per year for health services such as height/weight measurements and child vaccination. HPV SC is offered during these routine visits, which are conducted without a previous appointment. Owing to the large number of people each CHW is responsible for, scheduling visits by phone or other means is currently not feasible. If a woman performs SC at home, she is currently instructed to go to the health center within 30 days to retrieve her results, and if her results are positive, then she must have triage cytology at the health center [39].

### The Automated Messaging System

The Jujuy’s provincial program for the prevention of CC uses the National Screening Information System (SITAM, for its initials in Spanish) [7]. SITAM works as a Web-based screening registry that tracks all CC-related events of women screened in the public health system (screening, diagnosis, and treatment data). When a woman performs SC at home, the CHW collects the patient’s contact information and HPV sample data, which are entered into SITAM at the HPV provincial laboratory.

For the ATICA study, we developed a computerized messaging system, Automatic Messaging for Screening and Follow-up Care (MATYS, for its initials in Spanish), to send SMS messages to HPV-tested women and emails to CHWs. MATYS was linked to SITAM via an interface to access SC and Pap results, if any. Then, MATYS used these data to send a tailored SMS to the woman’s cell phone. Although MATYS is a one-way system and women were not expected to reply during the ATICA trial, MATYS registered any message sent by women, for the purpose of analysis.

### Participant Selection

Potential participants for the formative research stage were contacted by CHWs and were required to meet the following inclusion criteria: women had to be aged 30 years or older, should have performed HPV SC, should have the ability to read and write, and should be cell phone users.

### Data Collection

A total of six FGs were conducted using age (women aged 30-50 years, 40 years or older, and 51 years or older) and residence (rural or urban) as stratification criteria. Both variables are considered crucial in understanding the differences in cell phone usage [53,54]. FGs were conducted in Spanish by 2 female social science researchers: one acted as the moderator (VSA) and the other as the observer (MC); both of them neither lived in the Jujuy province nor had any relation whatsoever with the health care facilities or their authorities. The fieldwork was conducted in January 2018.

The FGs were conducted in locations that were easily accessible to the participants; no health care professionals or CHWs were present. Before each FG, the participants provided written informed consent to participate and to allow the audio recording of the discussions. Once the purpose of the study was explained to them, none of the participants refused to participate. Each FG lasted for an average of 2 hours.

### Analysis

The results of only five FGs are presented because participants of the another FG (FG4) did not meet the inclusion criteria. For the analysis, audio recordings from the FGs were transcribed and coded following the structural elements of the SMS. The women’s preferences and debates were coded to each element of the SMS (predefined themes): greeting line, message topic, sender, recipient, and closing line and persuasive phrase used as a cue to action (see Figure 2). This allowed the researchers to conduct a thematic analysis of the debates [55]. We used HBM constructs to explore the women’s beliefs, feelings, and opinions about the prevention of CC (SC and Pap) and how they linked their experiences with each SMS content. Atlas.Ti (version 7.5.4, ATLAS.ti Scientific Software Development GmbH, Berlin) was used for data processing. The transcripts were analyzed independently by 2 researchers (VSA and MC) to later compare, debate, and resolve the inconsistencies with the other members of the ATICA team.

The details of the methods and the results from the FGs are presented following the Consolidated Criteria for Reporting Qualitative Research [56].

The ATICA study’s protocol was registered in Clinicaltrials.gov (NCT03478397). In all its stages, including the formative research phase, ATICA study was approved by the Institutional Review Board of Center for Medical Education and Clinical Research, the Ethics Research Committee of the Jujuy Ministry of Health, the Institutional Review Board of the Harvard TH Chan School of Public Health, and the Deakin University Human Research Ethics Committee.

## Results

### Focus Group Characteristics

A total of 44 women participated in the five FGs. The majority resided in low-income urban areas in the capital of the province (San Salvador), 70% (31/44) had received secondary education or less, 75% (33/44) had public health coverage, and 90% (40/44) of the women shared their cell phones with their children and/or partners. In rural areas, cell phones were considered the home phone (FG5). In Table 1, a summary of the characteristics of the FGs is presented.

**Table 1:** Focus group participant characteristics.

Age (years)	Urban areas	Rural areas	Total
30-50	FG^a^1: 8 participants, majority completed their secondary school	FG2: 9 participants, majority completed their postsecondary school	17 women
51 or older	FG3: 9 participants, majority completed their primary school	FG4: Excluded from this analysis	9 women
40 or older^b^	FG6: 6 participants, majority completed their postsecondary school	FG5: 12 participants, majority completed their primary school	18 women
Total	23 women	21 women	44 women

^a^FG: focus group.

^b^Originally, four FGs were proposed. For deeper understanding, two additional FGs were conducted with women aged 40 years and older. In those cases, age segmentation was defined to obtain more data related to the use of technology.

### SMS Structure

#### Greeting

The women agreed that a simple “Hi” was unduly informal for an SMS. They considered that despite it being the characteristic tone of their relationship with the CHWs, the informal nature of the greeting line could discredit the whole message if included in the SMS. The option “Attention” was rejected for being too cold and inciting fear. It was only chosen by some women who would describe themselves as people who often hesitate going to a health center and to whom an imperative tone would “help” to cope with that hesitance (FG5). With regard to the option “Message from the health center,” women stated that even though it would help the reader understand the nature of the message, it was too distant to begin an SMS conversation in such a way. The option “Hello” was considered warm and formal.

#### Tone and Terms of Address

The preferences on the tone of the message and the terms of address were related to the issues that affected the overall content of the message. Therefore, women emphasized that the SMS should balance the formal nature of an institutional message “to transmit professionalism” (FG2) and the warmth intrinsic to their relationships with the CHWs.

With regard to the formal vs informal pronoun and verb conjugation use, the predesigned SMS cards using the formal “you” (“usted” in Spanish) were chosen. Furthermore, in the brainstorming activity, participants spontaneously proposed the use of the formal tone as well. For example:

[Spanish] Necesitamos **su** presencia en el centro de salud.

Author emphasis added

[Translation] We need your presence in the health center.

FG1: 30-50 years, urban area

#### Sender

In general, women considered that even though the message would be sent through MATYS, the sender of the SMS should mention a health authority as this would legitimize the content. In the women’s words, “It’s a message that isn’t just sent by anyone” (FG6).

After considering the different alternatives, “Health center” was the preferred option, instead of “Ministry of Health.” Participants mentioned that the Ministry of Health was an institutional figure, which they felt had little presence in their everyday life and, in some cases, was associated with negative past experiences. In the case of participants from the rural FG, they also added that the health center had geographic proximity which no other public health institution had (FG2 and FG3). Furthermore, most of the FG participants emphasized that the “Health center” was the CHWs’ reference institution, representing their relationship with CHWs, which, they stated, “it is founded on trust and familiarity” (FG2 and FG6):

...there’s a better relationship with the health care center. If you put “Ministry of Health” [in the SMS], maybe it’s like “Oh, it’s coming from San Salvador [the capital city],” but not really...

Woman 1; in a dismissive manner

...the difference between the city and the village...here what makes the difference is the closeness, we all know one another, it’s more familiar. The relationship with the professionals isn’t as distant as with the professionals in the city.

Woman 2; FG2: 30-50 years, rural area

#### Message Topic

The FGs included debates on how to mention the HPV test in the SMS. The debated options were “Results of the Human Papillomavirus test,” “Results of the HPV test,” “Results of the self-collection,” and “Examination results.”

In this regard, the private or shared use of cell phones was the main factor for diverging preferences. For example, women who shared their phone with other family members expressed privacy and confidentiality concerns and proposed avoiding the term HPV “to not worry their family” (FG5) and to avoid the social stigma surrounding HPV diagnosis (FG1). Some women mentioned that they would feel embarrassed should anyone find out that they had been tested; they expressed fear of the possibility of leaking private health information if a third party saw the SMS (FG1, FG3, and FG5).

Women who shared cell phones suggested using “self-collection” as an alternative to guarantee more privacy. Despite it being a technical term, women stated that they were familiarized with its use as it was the term CHWs used when offering HPV testing during home visits. “Self-collection” was considered more discreet than alternatives such as “human papillomavirus” or “HPV,” which not only explicitly named a stigmatized STI but were also confused with HIV and was difficult to comprehend for those with poor reading and writing skills. Furthermore, “self-collection” was more accepted than “Examination results” because the latter was considered too generic and would be confusing when trying to determine what the message was about. For example, some women joked, “Which of all the medical examinations I take [is the SMS referring to]?” (FG3).

#### Recipient

The advantages and disadvantages of SMS personalization by including the recipient’s name were also debated. Women from different FGs argued that using the recipient’s name was a safeguard against possible errors such as receiving an SMS meant for someone else. Moreover, including the name would be an indicator that it was a personal SMS, not a generic or mass message often sent by companies to their clients: “it’s an SMS sent to me, it’s not for anybody” (FG6).

Women with shared cell phones expressed the importance of including the name; otherwise, they would have no way of knowing if the SMS was for them or for another woman in the family. However, those who were against including the recipient’s name based their arguments on confidentiality issues: if a third party gained access to their phone, they would know that the woman had been tested.

The shared use of cell phones introduced a confidentiality issue. At the same time, women stressed that they needed help to get access to the information received through their cell phone, especially those who had vision problems, were not familiar with cell phones, and/or had poor reading and writing skills. In those cases, family members such as children or husbands helped them in the use of cell phones. This contradiction between confidentiality concerns and the need to ask for help to use a cell phone was pointed out by the moderator. To settle this contradiction, women highlighted that an SMS including the name of the recipient and using the term “self-collection” would take into account both concerns.

#### Closing and Cue of Action

For the closing sentence, FG participants discussed the different options proposed to encourage women to go to the health center. Participants found that phrases such as “We’ll be expecting you,” “We hope you can come to discuss the results,” and “Please visit your Health center” would have a persuasive effect. In the predesigned SMS to be sent to HPV-positive women, the phrase “It is important” was also included to emphasize the importance of contacting the health center. According to the women, this would help them anticipate an HPV-positive result. This was the option chosen by most participants:

Yes, because it is telling you to go to talk about it. I don't know...it’s like they’re softening the blow of...

There’s something odd, something’s wrong, so they want to talk about it.

Of course, that “let’s talk” means “the test came back positive.”

FG2: 30-50 years, rural area

Women also discussed variations of the SMS to be received by women who tested negative for HPV. The initial proposal of the SMS implied sending the actual negative test result via SMS. The proposed phrases were “You don’t have HPV. You must take the test again in 5 years” and “You don’t have the virus. You must take the test again in 5 years.” All these options were rejected across all FGs. According to the women, the SMS should promote contacting the health system to request information about their health in general and about HPV/CC in particular. In their opinion, receiving the HPV results via SMS could hinder the possibility of going to an appointment with the health care team and receiving information during a personal encounter:

What should not be included in the SMS?

Moderator

If it’s positive or negative

Woman 1

Not saying the result. Not even if it's negative?

Moderator

No, because when you go to the health clinic, the professional will explain it there.

Woman 1

It’s better if a professional tells you, in private. If your cellphone tells you “It’s negative…”

Woman 2

Then you wouldn’t go to learn more, it’s better to leave it unknown so you visit the health clinic.

Woman 1

To leave you with the idea that you have to go.

Woman 2

Right, because then a professional will explain it there.

Woman 3; FG1: 30-50 years, urban area

During the FGs, participants also suggested additional information that should be included in the SMS, such as regular hours of the health centers, the name of the specialist, and how to make an appointment. Thereafter, we explained why introducing this information would be unfeasible and might be confusing.

### The Draft Version of the Message

On the basis of the results of the FGs, study researchers produced a draft version of the SMS, as follows:

Dear [Woman’s Name].

The results of your self-collection are ready.

Please visit your health care center for a medical consultation.

It is important that you go.

This preliminary message to HPV-positive women based on the results from the FGs was presented to the provincial health authorities. In this draft version, “Dear” was used instead of “Hello” to respond to the women’s preferences regarding personalization and formality. The last sentence was used to emphasize the importance of them going to the health center, as a trigger to action.

### Validation and Pilot Test

The validation stage with the provincial health authorities did not present relevant divergences or inconsistencies in the results of the FGs with regard to the interpretation and understanding of the proposed message. However, they noted that “Dear” was not suitable for locals: “it is too formal and old fashioned.” As in the FGs, “Hello” was chosen as the best option.

In addition, the provincial health authorities pointed out that the phrase “Please visit your health care center for a medical consultation” could be understood by women in a restrictive manner. They could interpret that they should only see a physician. However, in health centers, very often Pap smears are taken by nurses or midwives. Therefore, the final version of the SMS excluded the term “medical.” Thus, the final version of this line was “Please visit your health care center for a consultation.”

Finally, the information suggested by the women about regular hours of the health centers to have a Pap smear or doctor’s name had to be dismissed. Provincial health authorities highlighted that this information was heterogeneous from one center to another and could therefore be confusing. Moreover, in case of a national scale-up of the intervention, these proposals would have to be dismissed as it was considered that it would be too complex to include this information for health centers of other provinces.

On the basis of the results of the FGs and the validation with the provincial health authorities, two final versions of SMS were designed: one for HPV-positive women and one for HPV-negative women. Neither SMS delivered the HPV test result. However, both SMS included the name of the recipient, used the term “self-collection,” and used formal language. The main nuance was in the intensity of the persuasive closing and cue to act. In the case of women with HPV-negative results, the SMS notified them of the availability of the result at the health center and encouraged them to retrieve them. In the message for HPV-positive women, the closing emphasized the importance of going to the health center for a checkup (see Tables 2 and 3).

Both SMS messages were validated by a survey with a sample of 14 women. This sample was taken in a hospital’s gynecological services waiting room (sample by convenience). Therefore, we excluded the CHWs’ mediation. The validation stage was useful for evaluating the understanding and wording preferences. The results of this stage showed that there were no important divergences or inconsistencies in the interpretation and understanding of the proposed message by women. As a result, new changes were not introduced.

In addition, a pilot test of the ATICA study’s intervention was conducted in July 2018. A total of 7 CHWs invited 36 eligible women to participate in the pilot test during their home visits. Once their HPV results were entered into SITAM, MATYS sent the corresponding SMS messages. Overall, 15 women did not receive the SMS because of errors in the procedure (eg, wrong cell phone numbers and errors in SITAM). In total, 21 eligible women received the SMS messages; of them, 18 (86%) were interviewed; all of them stated that they were highly satisfied with receiving the reminders via SMS.

**Table 2:** Final version of SMS contents for women who test negative for human papillomavirus.

Structural elements	Section
“Hello”	Greeting
“[Name]”	Recipient
“The results of”	Informative element
“your self-collection are ready.”	Message topic
“We’ll be expecting you”	Persuasive element
“in the health center”	Sender
“to retrieve them.”	Cue of action

**Table 3:** Final version of SMS contents for women who test positive for human papillomavirus.

Structural elements	Section
“Hello”	Greeting
“[Name]”	Recipient
“The results of”	Informative element
“your self-collection are ready.”	Message topic
“Please visit”	Persuasive element
“your health center”	Sender
“for a consultation.”	Cue of action
“It is important that you go.”	Persuasive element

## Discussion

### Principal Findings

This paper presents the results from FG debates to develop an SMS to increase the adherence of HPV-positive women to Pap-based triage, taking into account the women’s preferences regarding the content of the SMS. The study showed that women rejected receiving both negative and positive HPV results by SMS and stressed the importance of SMS content, highlighting the link they had with CHWs and the nearest health center. Women strongly preferred a personalized SMS; not using the word “HPV” was also a key component of the desired SMS content.

In our study, both women and health authorities were against receiving results via SMS, mainly because they considered that this type of news must be provided in a face-to-face health consultation so that the patients are able to ask questions and get rid of their doubts. The women’s rejection of receiving results by SMS was also mentioned in a study conducted in Chile, which found that Chilean women expressed concerns regarding the *impersonal nature* of the communication by SMS to communicate medical results [33]. Similarly, a colorectal cancer screening study in the United States found that patients did not want to receive results via SMS [57], particularly as it was not an appropriate communication mode to receive what they considered to be bad news. Our finding contrasts with a study conducted with low-resource women from Ohio, United States, which found that women preferred immediate phone calls to know the results (whether negative or positive) rather than waiting for an in-person medical consultation. In the Ohio study, providers favored withholding the HPV SC results as a strategy to bring women into the clinic [58] because they thought that the delivery of positive results over phone may scare women into denial and be a barrier to follow-up. Interestingly, this was not the reason put forward by women from our study, for whom it was mainly a question of not losing the opportunity to engage with the health system.

In our study, SMS messages were sent to women who were offered HPV SC by CHWs, community members with whom women have a relationship based on trust and familiarity [6,59]. Results from the FG discussions showed that this close link between women and the CHWs permeated the women’s choices regarding the greeting line, the overall tone of the SMS, and the selected sender. The need for messages to come from a trusted source has also been shown by studies analyzing mHealth interventions related to other health conditions in different contents [60,61].

The tone of the SMS was an important topic in the FG discussions related to the feeling of a close relationship between the sender and recipient. Women considered that the greeting line and message personalization were important to transmit the warm tone used by CHWs in face-to-face communication. In the same way, a study about blood pressure prevention highlighted the relevance of the SMS tone, wherein participants valued the polite tone of the text messages because it infused a sense of being recognized, respected, valued, and cared for [62].

The importance of a message reflecting the close relationship between women and the CHWs was also highlighted when discussing the sender. The health center, where CHWs are based, was the preferred message sender. Previous research in Jujuy also showed that CHWs are the key link between underscreened women and the health care system [6,59,63]. Similarly, one study on testing HIV among African immigrants in the United Kingdom observed that a trustworthy sender would be important to engage the recipient and avoid the SMS being deleted without a recipient reading it [60].

In text messaging–based health promotion interventions, a personalized SMS (eg, with the recipient’s name) has been associated with greater intervention efficacy [64]. A systematic review of behavior change interventions delivered by SMS messages showed that studies using personalized messages had higher follow-up care rates [65]. In our FGs, the inclusion of the recipient’s name was highly valued among Jujuy’s women. Similar to the aforementioned colorectal cancer screening study [57], our findings indicated that women might dismiss impersonal SMS messages as they may be interpreted as massive bulk texting.

However, several authors have noted that SMS delivered to shared cell phones to promote the treatment of STI presents the challenge of handling confidentiality [66-68]. In studies on STIs and SMS interventions, the main concern mentioned regarding confidentiality was the danger of disclosing results among relatives. In our study, confidentiality concerns mainly influenced how the HPV test was named in the SMS. The tension was between not mentioning the term “HPV,” which could increase the risk of misunderstanding, and including the term “HPV” in the SMS, which could result in breaking the women’s confidentiality. Thus, similar to findings from studies on HIV [35,38,69], Jujuy’s women preferred an indirect but understandable way to refer to the HPV test using the term “self-collection” and omitting “HPV” in the text message body.

Even though sharing a cell phone may be a confidentiality challenge, our study found that for many women, phone sharing was not necessarily an obstacle. On the contrary, in many cases, relatives acted as facilitators to help women access the information received through their cell phones. A similar finding was described in an HIV study in Uganda [38,70], where participants received their laboratory results by SMS, and if they had abnormal outcomes, they received a request to return to the health care center for treatment. The authors found that participants who had disclosed their HIV status to relatives and coworkers received help using the cell phone and/or reading the information in the SMS. The Ugandan participants with supportive and reliable networks could improve adherence by obtaining support to overcome barriers such as transport costs or asking for permission for time off work [38]. Therefore, to achieve a confidentiality balance in our study, we proposed including a woman’s name to personalize the SMS and using the term “self-collection” to indicate what the SMS was about.

### Limitations

The FG participants were recruited by the CHWs, which may have biased our sample selection. For example, some FG participants had another type of disease or a relative with a health problem (in two groups). This introduced a bias in group perceptions as they had a profound knowledge of certain health procedures. As we previously mentioned, the recruitment of one group failed: CHWs invited women without cell phones, and we were obligated to exclude this FG from this analysis.

Nevertheless, during the validation of the results by means of the survey, no significant discrepancies were presented in the FG findings. Another limitation is that the predesigned content used only affirmative phrases. A study on the promotion of colorectal cancer screening found a slightly higher effect in the groups that received invitations containing interrogative sentences than those that received declarative ones [71]. This modality should also be tested in future studies (eg, scaling-up evaluation). Despite the mentioned limitations, our approach of combining theory-informed content with user-driven feedback and local expert advice strengthens the potential of the SMS intervention.

### Conclusions and Implications

This formative research has shown women’s preferences with regard to greeting, mentioning the recipient, and the tone of the message (warmth and formality) to avoid the SMS from the health care system from being dismissed. The key terms of the SMS (in this case, the HPV test) must be carefully chosen in an endeavor to guarantee both confidentiality and comprehension of the content by the recipient.

Our findings have some implications for the design of mHealth interventions targeted at improving adherence to diagnoses and treatment of HPV-tested women. A personalized SMS may quickly notify the availability of HPV result; however, its content has to be carefully designed to transmit a health system’s proactive intention of caring for the population.

In our study, women preferred to not receive negative results via SMS because they believed that the communication between them and the health professionals during the delivery of the results should be prioritized. Although this was in agreement with other studies, it is necessary to determine if this preference is generalized to women from other settings.

## Abbreviations

ATICA: Application of Communication and Information Technologies to Self-collection (for its initials in Spanish)
CC: cervical cancer
CHW: community health worker
FG: focus group
HBM: health belief model
HPV: human papillomavirus
MATYS: Automatic Messaging for Screening and Follow-up Care (for its initials in Spanish)
mHealth: mobile health
NIH: National Institutes of Health
SC: self-collection
SITAM: National Screening Information System (for its initials in Spanish)
STI: sexually transmitted infection
